# Strengths and Limitations of BMI in the Diagnosis of Obesity: What is the Path Forward?

**DOI:** 10.1007/s13679-024-00580-1

**Published:** 2024-07-03

**Authors:** Katherine Sweatt, W. Timothy Garvey, Catia Martins

**Affiliations:** Department of Nutrition Sciences, University of Alabama at Birmingham, 675 University Blvd, Birmingham, AL 35294-3360 USA

**Keywords:** Obesity, BMI, Diagnostic, Screening, Adiposity, Anthropometrics

## Abstract

**Purpose of Review:**

This review aims to discuss strengths and limitations of body mass index (BMI) in diagnosing obesity, the use of alternative anthropometric measurements, and potential new technology that may change the future of obesity diagnosis and management.

**Recent Findings:**

The diagnosis of obesity requires the anthropometric assessment of adiposity. In clinical settings, this should include BMI with confirmation that elevated BMI represents excess adiposity and a measure of fat distribution (i.e., waist circumference (WC), waist to height ratio (WHtR), or WC divided by height^0.5^ (WHR.5R). Digital anthropometry and bioelectric impedance (BIA) can estimate fat distribution and be feasibly employed in the clinic. In addition, the diagnosis should include a clinical component assessing the presence and severity of weight-related complications.

**Summary:**

As anthropometric measures used in the diagnosis of obesity, BMI is generally sufficient if confirmed to represent excess adiposity, and there are advantages to the use of WHtR over WC to assess fat distribution. BIA and digital anthropometry have the potential to provide accurate measures of fat mass and distribution in clinical settings. There should also be a clinical evaluation for the presence and severity of obesity complications that can be used to stage the disease.

## Introduction

Obesity is a chronic disease defined as an abnormal or excessive fat accumulation which may impair health. Body mass index (BMI), defined as weight (kg) divided by the square of height (m) (kg/m^2^), is a simple anthropometric measure interrelating height and weight that is commonly used to identify the presence and severity of excess body fat in adults.

The World Health Organization (WHO) defines overweight as a BMI between 25 and 29.9 kg/m^2^ and obesity as a BMI greater than or equal to 30 kg/m^2^, further subdivided into class I (BMI = 30–34.9 kg/m^2^), class II (35–39.9 kg/m^2^), and class III (≥ 40 kg/m^2^) obesity. BMI is widely used in epidemiological research, as well as clinical settings. BMI is deeply embedded world-wide in research and clinical practice. It is used to make the diagnosis of obesity, as an indication for weight loss medications and bariatric surgery, and in guidelines for obesity management. The BMI calculation is also provided automatically in many electronic medical record systems and used at point-of-care in clinics by healthcare professionals. However, it is important to consider that BMI is only useful as a screening tool since is not a direct measure of adiposity and, as will be discussed, is unreliable as an indicator of the degree to which excess adiposity affects health in individual patients.

Waist circumference (WC) is generally recommended to assess fat distribution which adds information concerning cardiometabolic disease risk; however, waist to height ratio (WHtR), and WC divided by height^0.5^ (WHR.5R) [1] have been proposed as improved estimations of relative abdominal fat distribution. Other technologies that more directly assess FM content and distribution have also been advocated, although many of these may have limited feasibility for use in clinical venues. Additionally, 2- and 3-demisional optical (3DO) scanning technologies and digital anthropometry have been developed since the late 1980s and may provide a new direction for anthropometry that can be feasibly employed in clinics.

In this review, we discuss advantages and disadvantages of using BMI as the anthropometric component in the diagnosis and classification of obesity, as well as alternative indices of adiposity, and new technologies that may change the future of obesity diagnosis and management. The need to employ an additional clinical component in the diagnosis of obesity that reflects the impact of excess adiposity on health is also emphasized.

## BMI: Strengths and Limitations as a Diagnostic Tool

Adolphe Quetelet, a Belgian astronomer and mathematician, first proposed the Quetelet Index in 1859, as body mass (kg)/height (m^2^), as a measure of obesity [2]. The Quetelet Index was renamed Body Mass Index by Ancel Keys in 1972 who validated BMI against skin fold thickness. Ancel Keys was a polymath, nutritionist, and epidemiologist known for his Seven Countries Study, where he validated BMI and established a relationship between serum cholesterol and heart disease [3, 4].

The WHO adopted BMI for clinical classification of obesity in 1998 [5], and later added WC to assess central fat distribution and cardiometabolic disease risk. Soon after, the National Institute of Health (NIH) implemented BMI as the measure classifying obesity and interventional recommendations [6]. Subsequently, BMI cut-offs, shown in Table 1, were implemented to diagnose obesity and the clinical classification of severity of obesity. BMI is widely used in epidemiological and physiological research, by multiple health care organizations as guidelines for obesity management, in setting indications for weight loss medication by the FDA, and as the basis for diagnosis of obesity by clinicians.

**Table 1:** BMI, WC, and NICE cutoffs

	Caucasian		Asian		African American	
	Men	Women	Men	Women	Men	Women
BMI (kg/m^2^) (WHO)	Overweight: 25-29.9 Obese: ≥30		Overweight: 23-27.5 Obese: ≥27.5		Overweight: 25-29.9 Obese: ≥30	
Waist Circumference (WHO)	≥ 94 cm (↑ risk) ≥ 37 inches ≥ 102 cm (↑↑ risk ≥ 40 inches	≥ 80 cm (↑ risk) ≥ 31 inches ≥ 88 cm (↑↑ risk) ≥ 35 inches	≥ 90 cm ≥ 35 inches	≥ 80 cm ≥ 31 inches	≥ 94 cm ≥ 37 inches	≥ 80 cm ≥ 31 inches
NICE (degree of central adiposity based on WHtR)	0.5–0.59 (↑ risk) > 0.6 (↑↑ risk)		0.5–0.59 (↑ risk) > 0.6 (↑↑ risk)		0.5–0.59 (↑ risk) > 0.6 (↑↑ risk)	

Body mass index (BMI); World Health Organization (WHO), National Institute of Excellence in Health and Care (NICE) [7, 8]

The use of BMI for screening and diagnosis of obesity has many advantages. BMI is a quick, inexpensive, and reproducible measure useful in the initial screening for obesity. BMI correlates well with adiposity at the population level, as well as with cardiometabolic disease including T2D [9], gestational diabetes [10], atherosclerosis, stroke, and coronary artery disease [11]. There is a well-established relationship between obesity and osteoarthritis. The risk of knee osteoarthritis in particular increases 35% with every 5 kg/m^2^ increase in BMI [12]. Additionally, 40–70% of individuals with obesity have OSA [13, 14]. Epidemiological studies also indicate a relationship between obesity and some cancers, such as endometrial, esophageal adenocarcinoma, liver and kidney, however these data cannot establish a causal relationship [15]. Furthermore, many studies report increased risk of mortality and cardiometabolic disease once BMI increases over 25 kg/m^2^ [16]. The relationship between BMI and mortality is J-shaped, with overall mortality being lowest at a BMI between 22.5 and 25 kg/m^2^ and increasing by approximately 30% for every 5 kg/m^2^ increase in BMI above 25 kg/m^2^. Mortality is also increased below a BMI of 22.5 kg/m^2^ due in part to smoking and diseases causing cachexia [17].

BMI cut points have been developed addressing ethnic differences in adiposity and its relationship to cardiometabolic disease. Based upon evidence correlating BMI with risk of T2D in Asian-American adults, the American Diabetes Association recommends screening for diabetes when BMI ≥ 23 kg/m^2^ [18]. The body of evidence addressing this issue, including meta-analyses performed by the Working Group on Obesity in China, suggests that using a BMI cut-off of ≥ 23 kg/m^2^ would be the optimal single criterion for screening all Asian ethnicities for obesity based upon correlations with cardiometabolic risk factors and increased risk of mortality [19]. The WHO proposed BMI cut-offs of 23 kg/m^2^ for overweight and 27.5 kg/m^2^ for obesity in many Asian countries [7]. Other groups propose cut-offs of 23–24.9 kg/m^2^ for overweight and $\ge$25 kg/m^2^ for obesity based on risk for cardiometabolic disease in Asian countries [20].

While useful as a screening tool, BMI is not a direct measure of adiposity and cannot by itself be used to diagnose overweight or obesity. At the individual level, BMI lacks accuracy and reliability as an index reflecting adipose tissue mass. BMI overestimates adiposity in athletes with high muscle mass and in patients with edema, but underestimates adiposity in sarcopenic individuals with low lean mass. In addition, there are race and sex differences in the association between BMI and all-cause mortality, which are likely driven by differences in body composition, independent of BMI. People with a South Asian, Chinese, other Asian, Middle Eastern, Black African, or African-Caribbean family background are prone to central adiposity and their cardiometabolic risk occurs at lower BMI, justifying the use of lower BMI thresholds in defining overweight and obesity in these populations [21–23].

BMI does not provide an indication of the impact of excess adiposity on health in individual patients. Patients at any given BMI may or may not have obesity complication and related diseases. Individuals may have a similar BMI but differ in the amount and distribution of FM, thus having differing health outcomes [24]. Individuals with obesity who have no cardiometabolic disease risk factors are considered metabolically healthy, although these patients may still be at risk for biomechanical complications and should be followed longer term for the development of adverse outcomes [25, 26]. Due to the heterogeneous nature of obesity, further clinical assessment is needed to determine the degree to which excess adiposity affects an individual’s health.

The imprecision of BMI as a measure of adiposity weakens the association between BMI and cardiometabolic and other health risks and impairs its clinical use as a risk factor [27]. For example, BMI inadequately predicts cardiometabolic risk in those with sarcopenic obesity [28, 29], and mortality in the elderly is also more strongly predicted by low lean mass index than by BMI [30]. Therefore, a measure of lean mass and FM, not just BMI, is required in this population. A study by Liu et al. showed that obesity was a risk factor for sarcopenia defined by FM percent, however, when defined by BMI, obesity was protective [21].

BMI can be used as a screening tool for obesity but, for diagnosis, the BMI measurement must be clinically interpreted based on simple inspection or physical examination of the patient to confirm the presence of excess adiposity. Clinical assessment must then be used to identify those with a relatively low BMI but with excess adiposity, as well as those with high BMI but normal or low percent FM.

## Other Anthropometric Measures of Adiposity and Fat Distribution

### Waist Circumference

Excess fat accumulation in different depots can have significant implications for disease risk [11]. Relative distribution of fat to the visceral or intra-abdominal compartment is associated with insulin resistance, inflammation, and dysregulated secretion of adipokines, and is central to the pathogenesis of cardiometabolic disease [22, 23]. Similarly, the ectopic intracellular accumulation of fat in tissues, such as muscle and liver, is associated with insulin resistance and higher prevalence of metabolic syndrome. On the other hand, fat distribution to the periphery, such as upper and lower extremities and hips that occurs in subcutaneous fat depots is associated with increased insulin sensitivity and reduced prevalence of metabolic syndrome when adjusting for age and BMI [31–35]. For these reasons, assessment of WC as a measure of central adiposity is informative regarding cardiometabolic disease risk.

WC is complementary to BMI and is helpful in further assessing cardiometabolic disease risk. WC is a simple anthropometric measure of abdominal obesity and can be performed in a clinical setting as an estimate of visceral adipose tissue. Measurement of WC requires minimal personnel training and is best performed in a private setting using a tension-controlled tape measure placed around the patient just above the anterior superior iliac spine and horizontal to the floor. Without attention to standardized approaches, there can be a great deal of variation in the measurement of WC among individual personnel and clinics, diminishing its clinical value.

There is strong evidence that WC predicts mortality risk better than BMI [36, 37]. WC consistently and strongly predicts components of metabolic syndrome, T2D, CVD risk factors, and CVD events in cross-sectional studies and prospective cohorts. For CVD and all-cause mortality, WC is generally independent of, and a stronger predictor than BMI, even for lean individuals with BMI < 25 kg/m^2^ [38–40]. In combination with BMI, however, WC more precisely categorizes cardiometabolic risk in patients with overweight and obesity. As shown in Table 1, threshold values for WC indicating abdominal obesity and increased risk of T2D and CVD have been developed by multiple professional organizations and countries across the world for men and women.

Like BMI, there are important considerations in interpreting WC measurements. As shown in Table 1, there are ethnic variations in WC cut-points for predicting cardiometabolic disease, with lower thresholds for Asian populations [7, 19]. Cut-off values of WC are less meaningful at BMI ≥ 35 kg/m^2^, because most patients will exceed cut-off values independently of the presence or absence of insulin resistance or risk of cardiometabolic disease. However, while categorical cut-off values are commonly employed, the risk associated with WC is continuous. At any given BMI, the risk of T2D and CVD increases progressively with increasing WC even when the BMI exceeds 35 kg/m^2^ [41]. Lastly, WC alone has limitations. In addition to inter-individual variability in measurements and lack of standardized protocols, WC may penalize taller individuals because they have greater WC but may not be at greater cardiometabolic disease risk.

### Waist-to-Height Ratio

Ashwell et al. in 1996 proposed the waist-to-height ratio (WHtR) as the best anthropometric predictor of intra-abdominal fat, and the strongest predictor of cardiometabolic disease risk in adults. Furthermore, the distribution of the ratio between WC and height is similar between sexes, therefore WHtR does not require sex-specific cut points [42]. WHtR has been shown to be a better surrogate measure of body adiposity compared with other anthropometric indexes both in adults, children and adolescents [43], as well as a better predictor of visceral adipose tissue compared with WC, WHR and BMI [44, 45].

Ashwell et al. further proposed a ratio of 0.5 as a simple cut point to identify early risk of disease [46]. Systematic reviews and meta-analyses have shown that the WHtR is a better anthropometric measure for detecting hypertension in Asia-Pacific adults [47], superior to BMI in detecting CVD in both men and women [48], and superior to both BMI and WC in detecting cardiometabolic risk factors in men and women of various ethnicities [49].

The National Institute of Excellence in Health and Care (NICE) has recently recognized WHtR as an indicator of health risk, and encouraged “*everyone to keep their waist measurement to less than half their height to reduce the risk of potential health problems*” [50]. This report concluded that BMI is a practical measure of overweight and obesity but should be interpreted with caution because it is not a direct measure of central adiposity. In adults with BMI below 35 kg/m^2^, they advise to use WHtR, in addition to BMI, as a practical estimate of central adiposity and to help assess and predict health risks. The classifications (below) can be used for people with a BMI under 35 kg/m^2^ of both sexes and all ethnicities, including adults with high muscle mass.

- healthy central adiposity: WHtR 0.4 to 0.49, indicating no increased health risk.
- increased central adiposity: WHtR 0.5 to 0.59, indicating increased health risk.
- high central adiposity: WHtR 0.6 or more, indicating further increased health risk.

### Waist-Height − 5R

Nevill et al. in 2017 proposed a new WHtR, WC divided by height^0.5^ (WHT-5R) [1]. They showed this anthropometric measure to be a stronger predictor of a cardiometabolic disease risk composite score compared to WHtR, WC, BMI, waist-to-hip ratio, and body shape image analysis (in order from strongest to weakest). WHT-5R is the only waist ratio that is entirely independent of height and elements of adiposity (hip girth and BMI) and is the only anthropometric measure that does not penalize taller or shorter individuals because it removes the effect of height from WC. However, this study was done in a 90% Caucasian population and has not been validated in different ethnic groups.

Another study by Nevill et al. [51] compared the strength of the association between several anthropometric indexes (BMI, WHtR, WHT-5R, and WC) and four key cardiometabolic risk factors; HDL cholesterol, hemoglobin A1c (HbA1c), diastolic blood pressure (BP), and systolic BP. They concluded that all indices which include WC are superior to BMI in predicting cardiometabolic risk factors, but no single index was consistently superior in predicting BP, HbA1c, and HDL. The authors suggested that different anthropometric measures should be used depending on the clinical outcomes of interest. Nevertheless, WHT-5R has been proposed as a suitable index when screening for metabolic disease [52]. Further research is needed to determine which anthropometric indices are most appropriate in evaluating cardiometabolic disease risk factors after taking age, sex, race, and ethnicity into account.

### Body Shape Index

One criticism of WC and BMI is the inability to separate the impact of body shape (degree of central and peripheral adiposity) from body size (height and weight) on health. Krakauer and Krakauer in 2012 [53] proposed A Body Shape Index (ABSI) based on WC, weight, and height (WC/BMI^2/3^ x height^1/2^) to circumvent this limitation. A high ABSI score indicates that WC is higher than expected for a given height and weight, suggesting increased central adiposity.

ABSI has been analyzed in data from numerous countries in across the world, and a strong association between ABSI and mortality, particularly at higher ABSI levels, is consistently reported [53, 54]. ABSI outperforms WC and BMI in predicting all-cause mortality which could make it a useful anthropometric measure, particularly for population-level health surveillance and assessing community needs. ABSI is successful in predicting mortality associated with cardiovascular disease, chronic kidney disease (in men but not women), and some cancers highlighting the importance of body shape in the disease development [55–59]. Further research is needed to determine the usefulness of ABSI in a clinical setting, as well as sex and ethnic differences.

Central adiposity, measured as WC, has been shown to be a stronger predictor of overall cancer risk than BMI [60]. Similar findings have been reported for colon BMI and liver cancer [61, 62]. On the other hand, both general and abdominal obesity seem to be associated with osteoarthritis [63, 64]. A recent cumulative meta-analysis showed that neck circumference, BMI, WC and WHR are all associated with OSA in both Asians and Caucasians [65]. However, recent studies in Asian populations suggest that abdominal obesity, rather than general obesity, may play a more important role in OSA [66, 67].

## Body Composition and Fat Distribution

### Computed Tomography (CT), Magnetic Resonance Imaging (MRI) and Dual X-ray Absorptiometry (DXA)

Several methods are available that can measure body composition and FM distribution, as shown in Table 2. Direct measures of visceral adiposity and fat distribution can be obtained with CT and MRI. However, these methods are costly, require medical imaging equipment and trained technicians and, thus, are not feasible in a clinical setting. DXA provides data on body composition with detailed and fairly accurate measures of FM, fat-free mass (FFM), and bone mineral content (BMC). DXA also assesses fat distribution by measuring FM in limbs and trunk and can approximate distribution of fat to the visceral compartment. As is the case for CT and MRI, DXA is costly and requires a technician, and CT and DXA cannot be repeated frequently due to radiation.

**Table 2:** Advantages and limitations of anthropometric and body composition assessments

Method	Features Measured	Advantages	Limitations
**General adiposity**			
BMI*	- Body weight - Height	- Easy to calculate - Quick assessment - Low cost - Reproducible - Widely used - Useful for initial screening of obesity - Useful in population health studies - Correlates with health risks	- Indirect measure of adiposity - Cannot be used to diagnose overweight or obesity - No insight into fat mass content or distribution - Not suitable for certain populations - Influenced by height - Ethnic differences
**Anthropometrics for fat distribution**			
WC*	- Waist girth	- Quick assessment - Low cost - Consistent predictor of metabolic disease	- Requires trained personnel - Inter-individual variability in measurements - Lack of standardized protocols - Penalizes taller individuals - Ethnic and sex cut-offs
WHtR	- Height - Waist	- Quick assessment - Low cost - Strong predictor of cardiometabolic disease risk	- Requires trained personnel - Inter-individual variability in measurements - Lack of standardized protocols
WHT-5R	- Waist girth - Height	- Quick assessment - Low cost - Useful screening tool for cardiometabolic disease	- Requires trained personnel - Inter-individual variability in measurements - Lack of standardized protocols - Not validated across ethnicities
Body shape	- Height - Weight - Waist girth	- Quick assessment - Low cost	- Requires trained personnel - Inter-individual variability in measurements - Lack of standardized protocols - Not validated across ethnicities
**Digital Anthropometry**			
2D	- Body shape	- Quick assessment - No physical contact - Portable - Low cost - Patient can visualize obesity - Publicly available on smartphones	- Relationship between 2D results with cardiometabolic disease risk needs validation
3D	- Body shape	- Quick assessment - No physical contact - Low cost - Patient can visualize obesity	- Proprietary data - Algorithms not comparable between systems - Not easily portable - Relationship between 3D results and cardiometabolic disease risk needs validation
**Body Composition**			
MRI	- Total/regional FM - Skeletal muscle - Visceral adipose tissue - Ectopic fat	- High accuracy - High reproducibility - No exposure to ionizing radiation	- Costly - Requires trained personnel
CT	- Total/regional FM - Skeletal muscle - Visceral adipose tissue - Ectopic fat	- High accuracy - High reproducibility	- Costly - Requires trained personnel - Exposure to ionizing radiation
DXA	- Total/regional FM - Total/regional lean mass - Bone mineral content - Visceral adipose tissue	- Ease of use - Low radiation exposure - Accurate	- High equipment cost - Requires trained personnel - Radiation exposure
BIA	- Total, extracellular and intracellular water - FM and FFM -Visceral adipose tissue	- Ease of use - Low cost - Speed (fast)	- Population specific - Poor accuracy - Affected by edema
Air displacement plethysmography	- Total body volume - Total FM - Total FFM	- Relatively good accuracy - Speed (fast)	- Less accurate in disease - High equipment cost
3D photonic scanning	- Total/regional body volume	- OK for those with severe obesity - Ease of use	- Limited availability
Quantitative magnetic resonance	- Free and total body water - Total FM - Lean tissue mass	- Ease of use - Safety - Speed (fast)	- High equipment cost - Limited availability - Requires trained personnel
**Recommended Based on Clinical Feasibility and Importance**			
**BMI; WHtR; Digital anthropometry; BIA**			

FM: fat mass; FFM: fat free mass; * employ thresholds based on region, ethnicity, age, sex

### Bioimpedance

Bioimpedance analysis (BIA) is a noninvasive, quick, and relatively inexpensive method of body composition. Bioimpedance measure the electrical impedance of biological tissue to the flow of an alternating current at one or more frequencies applied to the skin surface through contact with electrodes [68]. Because conductivity of the tissues in the body differs, the impedance can provide estimations of body composition including FM, muscle mass, and body water, which are then used to estimate FM percentage and hydration levels.

Several BIA devices are available including systems with a single-frequency (SF-BIA), multiple frequencies-BIA (MF-BIA), and high-frequency current. Bioimpedance spectroscopy (BIS) uses a range of frequencies. Both SF-BIA and MF-BIA use population-derived equations to predict body composition, while BIS applies biophysical modeling to estimate body compartments. Reports have shown MF-BIA to better estimate changes in body composition following weight loss [69] and to be more accurate for patients with severe obesity, or abnormal hydration, compared to SF-BIA. However further research is needed to validate BIA in these cases.

Data suggests that BIA works well in healthy subjects and in patients with stable water and electrolytes balance with a validated BIA equation that is appropriate with regard to age, sex and race [70]. Both dehydration and overhydration, as well as changes in hydrational status over time, can impact BIA results. Strict adherence to fluid restriction at least 90 min before the measurement can be helpful in standardizing results [71]. The accuracy and reliability of BIA in individuals with obesity is controversial since BIA equations have been developed in normal-weight subjects. Many studies show that BIA underestimates FM in patients with obesity, sarcopenia and/or metabolic syndrome [72, 73]. Clinical use of BIA in subjects at extremes of BMI ranges, or with abnormal hydration, cannot be recommended for routine assessment of patients until further validation of accuracy in such conditions is completed. Longitudinal follow-up of body composition by BIA is possible in subjects with BMI 16–34 kg/m^2^ and normal hydrational status.

BIA is becoming widely used in obesity research and is being employed in clinical practice as well. This technology is useful when results are interpreted with an understanding of its limitations. BIA-derived FM is linearly associated with morbidity and mortality, compared with a U- or J-shaped association with BMI. Percent FM estimated from BIA is directly related to health outcomes such as cardiovascular disease, whereas both low and high BMIs are associated with increased risk of developing chronic disease [74]. Additionally, percent FA measured by BIA has been reported to be a better marker of cardiovascular disease risk than BMI in adults [75].

Advances in technology and further validation is necessary to understand the mechanisms behind the changes observed in acute illness, altered fat/lean mass ratios, extreme obesity, and body shape abnormalities.

## The Potential of Digital Anthropometry as a Robust Clinical Tool

Digital anthropometry is accessible, valid, reproducible, and cost-effective and provides robust anthropometric measurements of adiposity and fat distribution, which are associated with variable degrees of cardiometabolic disease risk. Furthermore, these technologies should be feasible for use in clinical care with respect to cost and logistics. Digital anthropometry using either three-dimensional optical (3DO) imaging systems or two-dimensional digital photography (2D DP) has recently been shown to provide noninvasive, accurate, and precise measurements of body composition [76].

**Fig. 1:**
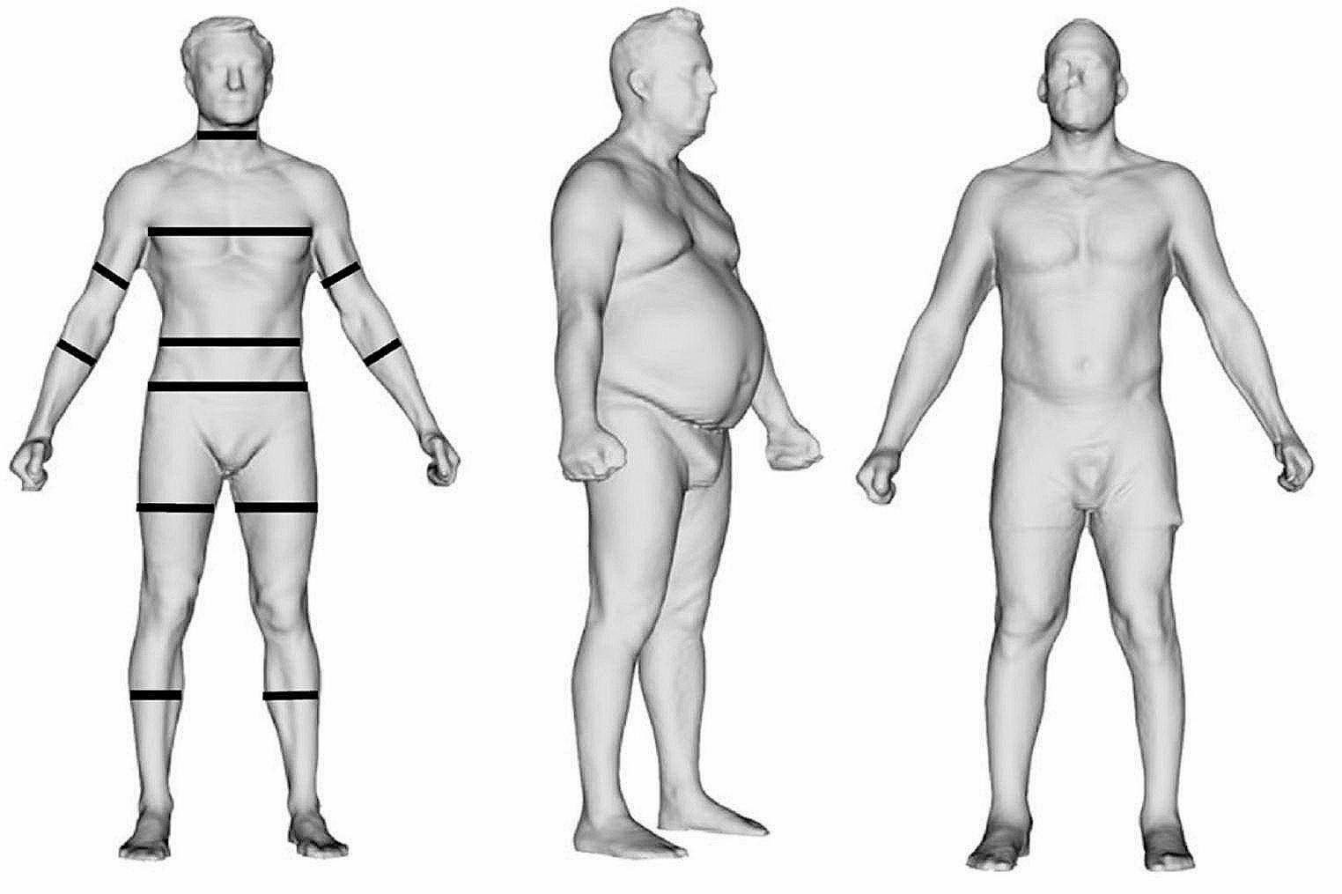
“e-tape” measurements of avatars from 3 different subjects. Taken from Minetto et al. [77] https://creativecommons.org/licenses/by/4.0/

3DO scanning captures external body shape and all systems commercially available follow a similar three-step process of data acquisition, processing, and anatomical measurements [76]. The scanners are relatively inexpensive, noninvasive, and there is no exposure to radiation. Data acquisition occurs through either structured light scanning, which evaluates light deformation patterns over subjects in view of the cameras, or time-of-flight scanning that measures the round-trip time for reflected photons to travel from the subject (wearing tight-fitted clothing) in the field of view to the image sensor and data is then used to create a three-dimensional point cloud that is adapted to obtain an avatar mesh of the human body. Lastly, anatomical measurements are obtained from the avatar using landmarking procedures allowing “e-tape” measures to extract multiple anatomical measurements, as demonstrated in Fig. 1 [77, 78].

Several studies have investigated the use of 3DO body shape to estimate body composition [79–83]. 3DO has been shown to accurately predict total FM [81] and total body composition (FM and FFM) [83]. However, in commercial 3DO scanners data is proprietary, and algorithms are not easily comparable between scanners. The measurements are also limited to linear, circumferential, volumetric, and surface areas. To circumvent these limitations, principal component analysis (PCA) can be used to produce detailed individual body shape models that predict body composition with greater accuracy than traditional anthropometric measurements and can be used across all scanning systems. Additionally, PCA of 3DO scans improves prediction of cardiometabolic risk factors such as blood lipids and T2D markers [83].

Digital imaging has become even easier to implement with the widespread public availability of smartphones with high quality cameras enabling acquisition of 2D DP. 2D DP has been validated to estimate total and abdominal FM in adults [84] and overcomes many of the limitations of 3D imaging systems which can entail large equipment that is cumbersome to transport. Applications are available for smartphones (e.g., Fit.Your.Outfit) that guide an operator on how to obtain high-quality digital images for analysis. The image is uploaded to the Cloud-based neural network educated by deep learning machine technology. The human profile is then extracted and conditioned as a single, homogenous, white pixel bitmap silhouette on a black background. The percentage of white versus black pixels is used to compute a real pixel size, and proprietary equations are used to compute total body and abdominal FM. Once the image is snapped, the estimation of body composition is approximately 12 s with high speed WIFI connection [84].

This new technology fulfills the need of a practical and feasible method to combine whole- and regional body composition assessment for reliable, cost-effective screening of individuals for risk of cardiometabolic disease without the technical error of the manual measurement of regional girths. 2D DP sets the stage for future machine learning opportunities for relating body shape and composition to other clinical risk factors for chronic disease [34], without the limitations of 3DO digital imaging systems.

Digital anthropometry serves as another means to communicate concerns related to excess adiposity, as numerical descriptors of body size, such as BMI and WC, are better understood with visual analysis of body shape [77, 85]. Minetto et al. makes the point that people are inherently more interested in how they look to the eye than in numerical descriptors of their body size and composition [77]. Importantly, digital anthropometry can be used to demonstrate the longitudinal effectiveness of therapy and assess the quality of weight loss regarding fat distribution and loss of lean versus FM. While the potential for clinical application is clear, the major current limitation to the application of digital anthropometry is that more studies are needed validating the association of body shape phenotypes with cardiometabolic disease risk factors and outcomes in different populations.

## Beyond BMI: The Need for a Clinical Component in the Diagnosis Indicating Health Status

Obesity adversely affects quality of life and increases the risk of developing obesity complications and related diseases including type 2 diabetes (T2D), cardiovascular disease (CVD), obstructive sleep apnea (OSA), and some cancers [86]. These complications confer disease-related morbidity and mortality and impair quality of life. The diagnosis of obesity based on BMI per se provides insufficient indication of the impact of excess adiposity on health in individual patients. In this context, it is important to consider that the burden of complications varies greatly among individuals at any given BMI [87, 88].

The need for both an anthropometric component to the diagnosis of obesity and a clinical component that reflects impact on health is consistent with a complications-centric approach to care in which the treatment and prevention of complications becomes the primary end point of weight loss therapy, not the loss of a given number of kilograms body weight per se [89]. There is now harmonization of clinical guidelines around complications-centric care beginning with American Association of Clinical Endocrinology (AACE) [90], European Association for the Study of Obesity (EASO) [91], the Australian [92] and Canadian [93] guidelines and those advocated by the Obesity Medical Association (https://obesitymedicine.org/obesity-algorithm/). The AACE guidelines explicitly call for both an anthropometric component (e.g., BMI) and a clinical component to the diagnosis; the latter representing surveillance for risk, presence, and severity of weight related complications based on a standard intake exam, obesity-focused history, and review of standard laboratory tests. This information can be used to stage disease severity as a second step. Staging approaches for obesity include among others: (i) a simple approach advocated in the AACE obesity guidelines [10] with stage 0 indicating no complications, stage 1 having one or more mild-moderate complications, and stage 2 at least one severe complication; (ii) the Edmonton System [94] that assesses medical, functional, and psychological impact of obesity over 5 stages of severity; (iii) an approach advocated by EASO [95] that includes the 3 dimensions of multifactorial etiology, the degree of adiposity, and specific health risks gradated from low, to intermediate, to high. An ongoing Lancet Commission on the Definition and Diagnosis of Clinical Obesity will address many of these issues [96].

The need for a conceptualization of obesity that goes beyond BMI is addressed by a new medically actionable diagnostic term for this disease, namely *Adiposity-Based Chronic Disease (ABCD)*, which has been endorsed by both the AACE [97] and the EASO [98]. This term indicates what we are treating (abnormalities in the mass, distribution, and function of adipose tissue) and why we are treating it (a chronic disease that gives rise to complications that impair health). As a diagnostic term, ABCD implicates the need for an evaluation of health risks and complications that can subsequently be used for disease staging.

## Conclusion

In conclusion, BMI is widely used around the world for clinical and research applications and is automatically calculated in electronic medical records to assist in point-of-care clinical decisions. Nevertheless, BMI should only be used as a screening tool. Elevated values of BMI should be confirmed to represent increase adipose tissue mass by inspection and physical examination of individual patients. Moreover, BMI is only an indirect measure of adiposity, does not assess body composition, and is not sufficient to indicate the degree to which increased adiposity affects health in individual patients (i.e., the presence and severity of obesity complications).

Measurement of WC adds a measure of central adiposity and is a risk factor for cardiometabolic disease outcomes independent of BMI. The relationships between BMI, WC values, and cardiometabolic disease risk varies as a function of sex, race, ethnicity, age, and fitness level among other factors. Indices that incorporate WC and height such as WHtR or WHT-5R, outperform both BMI and WC for prediction of several cardiometabolic risk factors and outcomes. The advantage of WHtR is that the same cut-off values can be used regardless of sex, muscle mass, and race/ethnicity. In any event, measurements of WC require trained personnel and application of a standardized approach.

More sophisticated tools can provide accurate measures of body composition and fat distribution, including DXA, CT, and MRI. However, these technologies are expensive and cannot feasibly be employed in clinical settings. Two technologies have emerged that can feasibly be used to provide useful estimations of FM content and distribution for informed clinical decisions: BIA and digital anthropometry. These methods are currently under-utilized but can be recommended for wider application in the future for more precise estimations of adiposity and body composition. With the advent of second-generation obesity medications such as semaglutide and tirzepatide [99], which can produce degrees of weight loss overlapping with those observed following bariatric surgery, measures of body composition will become more imperative in the evaluation of patients at baseline and the quality of weight loss in response to therapy.

All these considerations pertain to the anthropometric component of the diagnosis of obesity. The clinical component of the diagnosis, consisting of an evaluation of the risk, presence, and severity of obesity-related complications, remains essential for evaluating the impact of adiposity on health. Both anthropometric and clinical components are needed as the basis of a complications-centric approach to care within the conceptual framework of the diagnostic term ABCD.

## Data Availability

No datasets were generated or analysed during the current study.
