# Correction: Strengths and Limitations of BMI in the Diagnosis of Obesity: What is the Path Forward?

**DOI:** 10.1007/s13679-024-00584-x

**Published:** 2024-08-13

**Authors:** Katherine Sweatt, W. Timothy Garvey, Catia Martins

**Affiliations:** Department of Nutrition Sciences, University of Alabama at Birmingham, 675 University Blvd, Birmingham, AL 35294-3360 USA

**Correction: Current Obesity Reports (2024) 13:584–595**

10.1007/s13679-024-00580-1

The original version of this article unfortunately has error. In Table 2, second column (Features Measured), as follows:

-In the WHtR line, it should be waist, NOT weight. Therefore, in Table it should be both Height and Waist.

-In the WHT-5R, height should be added to waist. Therefore, in Table it should be both Height and Waist.

The original article has been corrected.

